# Sex-specific impact of vitamin D and B9 concentrations on neuroticism: a polygenic score-based study

**DOI:** 10.3389/fpsyt.2026.1788663

**Published:** 2026-04-24

**Authors:** Margarita Alfimova, Vera Golimbet, Ekaterina Semina, Yulia Chaika

**Affiliations:** Mental Health Research Center, Moscow, Russia

**Keywords:** folate, neuroticism, polygenic scores, risk factors, vitamin D

## Abstract

**Introduction:**

Neuroticism is a personality domain with prognostic value for physical and mental health. To properly inform public health policy, it is crucial to uncover the mechanisms underlying high neuroticism. Many internal and external factors that affect brain development and functioning and therefore might contribute to the variability of neuroticism remain understudied. Among them, the impact of vitamin sufficiency is of great interest, as it is a modifiable factor. This study aimed to evaluate the associations of neuroticism with vitamin D (VD) and vitamin B9 (VB9) using polygenic scores (PGS) in a nonclinical cohort.

**Methods:**

We analyzed data from 348 healthy unrelated individuals, including neuroticism scores on the Eysenck Personality Inventory, VD-PGS, VB9-PGS and PGS for neuroticism-related traits.

**Results:**

The analysis controlling for demographic and genetic confounders revealed a negative association between VB9-PGS and neuroticism scores in women and a positive association between VD-PGS and neuroticism scores in men. The highest values of the VD-PGS were observed in men, who scored high on both neuroticism and extraversion. In men, unlike women, neuroticism scores were not correlated with PGS for neuroticism but were associated with PGS for bipolar disorder type 1 and alcohol use disorders.

**Conclusion:**

The results suggest that the effects on neuroticism of genetic propensity for suboptimal vitamin D and B9 concentrations might differ across the two sexes. The findings are consistent with the idea of the importance of vitamin B9 for emotional stability in women and indicate the involvement of genetic factors predisposing to higher vitamin D levels in excitability-related components of neuroticism in men.

## 1 Introduction

Neuroticism is a basic personality domain with prognostic value for physical and mental health (1, 2). It represents individual differences in emotional stability, i.e., a tendency to experience a wide range of negative emotions in response to various types of stress. According to the Big Five personality model, neuroticism consists of multiple facets, including anxiety, angry hostility, depression, self-consciousness, impulsiveness, and vulnerability. Epidemiological research has shown associations of neuroticism and its facets with engagement in unhealthy behaviors, e.g., smoking (3), as well as with depressive and anxiety disorders (4, 5), addictions (6), and mortality risk (7, 8).

Individual differences in neuroticism are partly due to genetic influences, with heritability estimates of approximately 40% (9). Genome-wide association studies (GWAS) have identified biological pathways related to this trait, such as “neurogenesis” and “neuron differentiation”. In accordance with the conceptualization of neuroticism, one of the most significant and reproducible associations was found in the *CRHR1* gene locus encoding the corticotropin-releasing hormone receptor, an important player in the stress response. In line with epidemiologic research, high genetic correlations of neuroticism with anxiety, depression, and irritability symptoms were obtained (10–13).

At the same time, more than half of the phenotypic variance in neuroticism is explained by environmental influences (9). Managing them might reduce neuroticism and health risks. However, the environmental causes of high neuroticism have traditionally received less attention than genetic ones, with an emphasis placed on social aspects of life (14–16). A wide range of internal and external factors that affect brain development and functioning and therefore might contribute to the variability of neuroticism remain understudied. Among them, the impact of vitamin sufficiency is of great interest, as it is a modifiable factor.

One of the most promising candidates is vitamin D. Vitamin D is synthesized mainly in the skin from 7-dehydrocholesterol by ultraviolet rays (vitamin D3) and comes from food (vitamin D2). Both compounds undergo two stages of hydroxylation. The first one occurs in the liver and results in calcidiol (25(OH)D), the major circulating form of vitamin D. At the second stage occurring in the kidneys, the active hormone calcitriol (1,25(OH)2D) is produced (17). Vitamin D deficiency is common in many countries. Its prevalence reaches 40% in Europe (17), 68% in the most urban district (Colombo) of Sri Lanka (18), and 56% in the majority of geographic regions of Russia (45–70° north latitude) (19).

Vitamin D has long been known to play a role in bone metabolism. The recent discovery of receptors for vitamin D, along with enzymes that synthesize and metabolize it, in the brain has led to the recognition of vitamin D as a neurosteroid involved in neurodevelopment, neuroplasticity, and neuroimmunomodulation and has raised the question of its role in mental health phenotypes (20, 21). Although the data are somewhat inconsistent, emerging evidence suggests that vitamin D status is associated with phenotypes for which neuroticism is a very strong correlate, including depressive and anxiety disorders (17, 20, 22, 23).

Vitamin B9 (folate), obtained from food, is a cofactor in single-carbon transfer reactions in the biosynthesis of many compounds. The association between vitamin B9 status and mental health is thought to be due to its important role in methylation, as well as the catecholamine synthesis-inhibiting and neurotoxic effects of homocysteine, whose levels increase with folate deficiency (24–26). Like other B vitamins, vitamin B9 has neuroprotective effects and, among mental disorders, has attracted the greatest interest in connection with neurodegeneration and cognitive decline (27, 28). Recently, as with vitamin D, correlations of folate concentrations with depression and anxiety have been identified (24, 26, 29). However, the impact of vitamin D and B9 supplementation on depression and anxiety remains controversial, and it cannot be ruled out that these disorders are a cause rather than a consequence of vitamin deficiency (29, 30).

The up-to-date approach to examine causality between modifiable risk factors and outcomes of interest is Mendelian randomization (MR). In addition, GWAS-derived polygenic scores (PGS, also called polygenic risk scores) can be applied to estimate an effect of liability to an exposure on an outcome. PGS may serve as instrumental variables in MR under certain conditions and are especially useful in the case of smaller sample sizes (31). MR- and PGS-based studies of the effects of individual differences in vitamin D and B9 concentrations on depression and anxiety have yielded mixed results (25, 32–36). To the best of our knowledge, MR- and PGS-based investigations of the vitamin B9 contribution to neuroticism variability are lacking, and only a few studies on vitamin D exist. Specifically, Jiang et al. (37) conducted an MR-study and reported no effect of the 25(OH)D concentration on neuroticism. In contrast, using PGS for 25(OH)D concentration (hereinafter VD-PGS), Avinun et al. (38) revealed an association between decreased vitamin D levels and increased neuroticism. Thus, the role of vitamin D and B9 deficiency in neuroticism variability remains uncertain. Given that the levels of vitamins are determined by individual metabolic characteristics in interaction with the geographical and cultural aspects of lifestyle, their role needs to be explored in different populations.

The aim of the present study was to evaluate the associations of neuroticism with the VD-PGS and VB9-PGS in a nonclinical sample of Russians. We expected to find negative correlations of neuroticism with the VD-PGS and VB9-PGS. The hypothesized directionality of associations was based on prior research demonstrating correlations of low folate and vitamin D levels with an increased risk of depression and anxiety (17, 23, 24).

## 2 Materials and methods

We studied data from 348 healthy unrelated Russian individuals (aged 16–65 years; 62% women) from the institution-based Biomaterial Collection Fund “NeuroResource” (39). All procedures were carried out in accordance with the Declaration of Helsinki. The local ethical board approved the procedure of DNA biobanking and the collection of supplementary information, as well as their subsequent use for research purposes (no. 98 dated 11 September 2007). The participants provided informed consent at the recruitment stage. Neuroticism scores and GWAS-derived PGS for biological, psychological and clinical traits were available for each subject. Neuroticism was assessed with the Eysenck Personality Inventory, which consists of the Neuroticism, Extraversion and Lie scales. The Neuroticism scale includes 24 items with answers in binary format (yes/no). Extraversion is assessed similarly. The genome-wide data for PGS were obtained using HAP610-QUAD and GSA v2/v3 Bead-Chips and were prepared as described previously (40). For the main analysis, individual PGS for the three phenotypes of interest were computed by the Michigan Imputation Server (41) using the following UK Biobank based scores from the Polygenic Score Catalog (42): PGS000882 developed by (34) for VD-PGS, PGS001153 (43) for VB9-PGS, and PGS002708 (44) for neuroticism PGS (N-PGS). For auxiliary analyses, PGS for bipolar disorder types 1 and 2 (BD1, BD2) (45), attention deficit hyperactivity disorder (ADHD) (46), alcohol use disorders (AUD) (47), and major depressive disorder (MDD) (48) calculated with LDpred2 (49) were applied.

Statistical analyses were conducted with JASP (50). A t-test was used to compare men and women by age and variables of interest, as well as to compare PGSs between genotyping platforms. Pearson correlations were calculated to examine relationships between genetic variables. The main analysis involved building two regression models: one included VD-PGS as a predictor of neuroticism, and the other included VB9-PGS as a predictor of neuroticism. To build each model, we used a hierarchical approach, with a null model containing biological sex, age, two ancestry-related principal components (PCs), and N-PGS. PGS of one of the vitamins was added to the null model, and the change in the model parameters (*ΔF* and *ΔR^2^*) was estimated. Since the data were tested for compliance with the hypothesis, no correction for multiple testing of the study hypothesis was made. The significance level was set at *p* = 0.050. A similar analysis of extraversion was performed to explore the specificity of the patterns identified for neuroticism.

Several auxiliary analyses were run. First, two regression models were built to assess the effects of the VD-PGS and VB9-PGS on neuroticism in men and women separately. Again, we used the hierarchical approach. The null model included age, two PCs, and N-PGS. To minimize the multiple testing problem, we added VD-PGS and VB9-PGS to the models simultaneously. Then, to compare the regression coefficients for VD-, VB9- and N-PGS between men and women, three additional regression analyses were conducted. Each analysis included sex, PGS for a trait of interest and an interaction term of sex with the PGS as predictors of neuroticism. Next, to compare groups with different combinations of neuroticism and extraversion (four “temperaments”) on VD-PGS, we applied an analysis of covariance (ANCOVA), controlling for age and two PCs. Finally, to address pleiotropic effects of the polymorphisms included in the VD-PGS on phenotypes reflecting a tendency toward mood instability, excitability, irritability, and anger, partial (conditioned on the two PCs) Pearson correlations of VD-PGS with PGS of several relevant phenotypes were calculated.

## 3 Results

### 3.1 Sample characteristics

The sample characteristics are presented in **Table 1**. The correlation coefficients between the PGSs used in the regression analyses are given in **Table 2**. Women had higher neuroticism scores (*t* = 4.96; *p* < 0.001; Cohen’s *d* = 0.55) and lower VD-PGS (*t* = 2.20; *p* = 0.028; Cohen’s *d* = 0.24) compared to men. There were no significant correlations between the PGSs. The PGSs did not differ depending on genotyping platform.

**Table 1:** Sample characteristics.

Variable	Men (*n* = 132)	Women (*n* = 216)	All (*n* = 348)
Age (years)	31.61 ± 11.95	30.97 ± 11.86	31.22 ± 11.88
EPI N***	10.33 ± 4.52	12.99 ± 5.04	11.98 ± 5.01
EPI E	12.14 ± 3.72	11.35 ± 4.11	11.65 ± 3.98
N-PGS	0.14 ± 1.02	-0.003 ± 0.97	0.05 ± 0.99
VD-PGS*	0.12 ± 1.02	-0.12 ± 0.99	-0.03 ± 1.01
VB9-PGS	0.09 ± 1.01	0.14 ± 0.97	0.12 ± 0.98

PGS, polygenic scores; VD, vitamin D; VB9, vitamin B9; N, neuroticism; E, extraversion; EPI, the Eyesenck Personality Inventory. PGS are estimates standardized using the mean and standard deviation of the normative group of healthy Russian individuals, *n* = 760 (40). Significant differences between men and women: *** - *p* < 0.001, *- *p* < 0.05.

**Table 2:** Pearson correlations between genetic variables.

Variable	N-PGS	VD-PGS	VB9-PGS	PC1
VD-PGS	-0.03			
VB9-PGS	0.05	-0.03		
PC1	-0.03	-0.01	0.05	
PC2	-0.03	-0.04	0.08	0.41***

PGS, polygenic scores; VD, vitamin D; VB9, vitamin B9; N, neuroticism; PC, ancestry-related principal component. *** – *р* < 0.001.

### 3.2 Associations of PGS with neuroticism in the whole sample

The main analysis revealed a significant positive association of neuroticism with the VD-PGS (**Table 3**). Female sex (*β* = 2.94 ± 0.54; *p* < 0.001) and higher N-PGS (*β* = 0.67 ± 0.26; *p* = 0.011) were also significant predictors of higher neuroticism. The negative correlation between VB9-PGS and neuroticism did not reach the significance threshold. Neither VD-PGS nor VB9-PGS predicted extraversion (**Table 3**). Notably, in contrast to neuroticism, extraversion was not associated with the N-PGS (*β* = 0.25 ± 0.22; *p* = 0.24), and the association between VD-PGS and neuroticism remained significant when N-PGS was excluded from the null model (*p* = 0.018).

**Table 3:** Association of neuroticism and extraversion with polygenic scores for vitamin D and B9 levels.

Predictor	Outcome	*β ± SE*	*ΔF*	Δ*R^2^*	*p*
VD-PGS	EPI N	0.64 ± 0.26	6.16	0.016	0.013
VB9-PGS	EPI N	-0.38 ± 0.27	2.08	0.006	0.150
VD-PGS	EPI E	0.09 ± 0.21	0.19	0.001	0.667
VB9-PGS	EPI E	-0.15 ± 0.22	0.49	0.001	0.483

PGS, polygenic scores; VD, vitamin D; VB9, vitamin B9; N, neuroticism; E,extraversion; EPI, the Eyesenck Personality Inventory. The main results of four regression analyses are presented. *β,* unstandardized regression coefficient*; SE*, standard error; Δ*R^2^* is an increment in the explained variance of personality trait compared to a null model that included sex, age, two ancestry-related genetic components, and N-PGS; *ΔF* and *p* are a respective increment in *F* and its *p*-value.

### 3.3 Sex-specific relations of PGS with neuroticism

**Figure 1:**
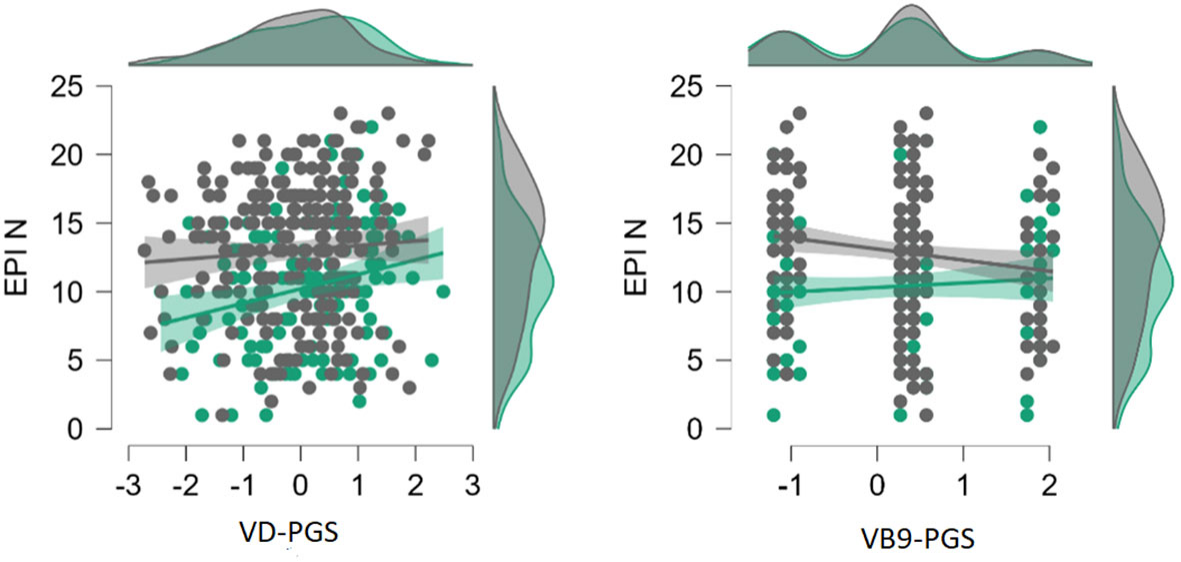
Associations of neuroticism with polygenic scores for vitamin D and B9 concentrations in women (gray) and in men (green). PGS, polygenic scores; VD, vitamin D; VB9, vitamin B9; N, neuroticism; EPI, the Eyesenck Personality Inventory.

Given the substantial contribution of sex to the manifestations of neuroticism and the direction of the VD-PGS effect on neuroticism being opposite to the hypothesis, auxiliary analyses were conducted to aid interpretation of the findings. First, the effects of the VD-PGS and VB9-PGS on neuroticism were assessed separately in men and women (**Figure 1**). In women, VD-PGS did not affect neuroticism (*β* = 0.35 ± 0.34; *p* = 0.312), whereas VB9-PGS (*β* = -0.80 ± 0.35; *p* = 0.025) and N-PGS (*β* = 1.04 ± 0.35; *p* = 0.003) did. In men, the pattern was opposite: there were no effects of VB9-PGS (*β* = 0.41 ± 0.40; *p* = 0.304) or N-PGS (*β* = 0.18 ± 0.39; *p* = 0.636), whereas VD-PGS was significantly positively associated with neuroticism (*β* = 1.11 ± 0.39; *p* = 0.005). In the regression models aimed to compare the regression coefficients for VD-, VB9- and N-PGS between men and women, the interaction term of sex with the VB9-PGS was significant (*p* = 0.036), while those of sex with the VD-PGS and N-PGS did not reach the significance threshold (*p* = 0.173 and *p* = 0.084, respectively).

**Figure 2:**
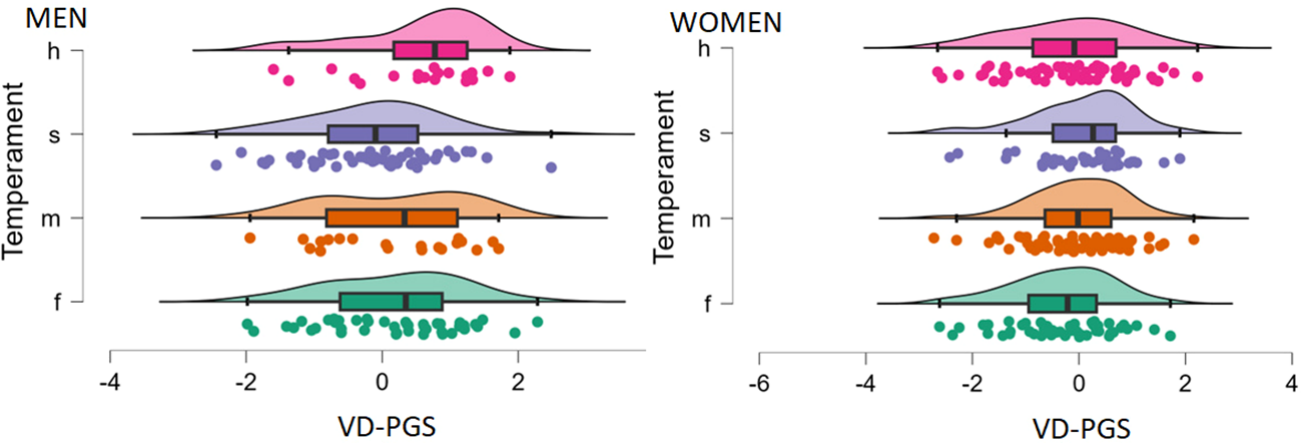
Polygenic scores for vitamin D concentration (VD-PGS) depending on neuroticism x extraversion pairing (temperament) and sex. Temperaments: m - melancholic, f - phlegmatic, s - sanguine, h – choleric.

We continued to explore the identified association between VD-PGS and neuroticism considering the multifaceted nature of this personality domain. Studies of the Big Five personality model have shown the presence of two nonorthogonal factors in the structure of neuroticism, namely, the Withdrawal (depression, vulnerability, and anxiety) and Volatility (emotional instability, angry hostility, and impulsiveness) factors (51). In Eysenck’s model, the content of these factors echoes descriptions of traits resulted from the combinations of high neuroticism with low and high extraversion, respectively (52). With low extraversion, high neuroticism manifests as moodiness, anxiety, rigidity, and sobriety (“melancholic temperament”), which corresponds to the Withdrawal factor. The pairing of high neuroticism with high extraversion results in touchiness, restlessness, aggressiveness, and excitability (“choleric temperament”), which corresponds to the Volatility factor. Since these factors may have distinct biological sources (51), we compared groups of men and women with different combinations of neuroticism and extraversion scores (four “temperaments”) on VD-PGS (**Figure 2**). Among men, the VD-PGS tended to be higher in those scoring high in both neuroticism and extraversion relative to the other subgroups: *F* = 2.51; *p* = 0.061; η²_p_ = 0.057. The *post hoc* Tukey test showed that this subgroup differed significantly from men with low neuroticism and high extraversion: *p* = 0.039; Cohen’s *d* = 0.711. No significant differences were found in women.

**Figure 3:**
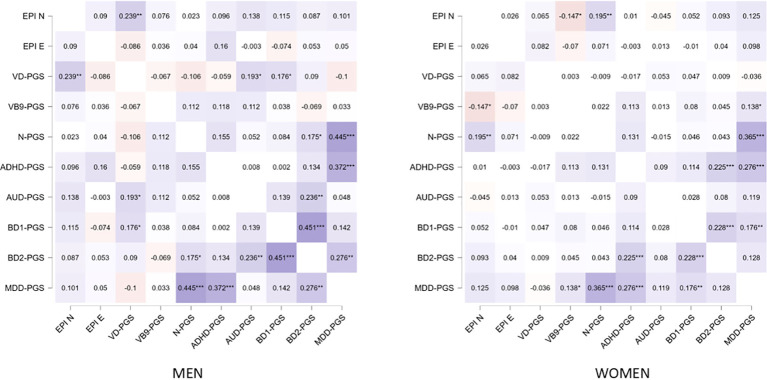
Heatmap of partial Pearson’s product-moment correlation coefficients between personality and polygenic scores for disorders and vitamin concentrations. The correlations are conditioned on the first two ancestry-related principal components. Violet squares mean negative correlations; orange means positive correlations; darker colors correspond to larger values. Significance: *** - *p* < 0.001, ** - *p* < 0.01, *- *p* < 0.05. PGS, polygenic scores; VD, vitamin D; VB9, vitamin B9; EPI N and E, neuroticism and extraversion scores on the Eyesenck Personality Inventory; ADHD, attention deficit hyperactivity disorder; AUD, alcohol use disorders; BD, bipolar disorder; MDD, major depressive disorder.

Finally, we tested whether the association of VD-PGS with neuroticism in men could result from pleiotropic effects of the polymorphisms included in the VD-PGS on phenotypes reflecting a tendency toward mood instability, excitability, irritability, and anger. For this analysis BD1, ADHD and AUD were chosen since the traits comprising the Volatility factor are highly characteristic of each of these disorders (53, 54). The BD2-PGS and MDD-PGS were used as a control because BD2 and MDD symptoms are more compatible with the Withdrawal factor. Correlations of VD-PGS with PGS for these phenotypes were explored as a proxy for genetic correlations. The analysis revealed that in men, the VD-PGS was significantly positively correlated with the BD1-PGS and AUD-PGS (**Figure 3**). There were no significant correlations in women.

## 4 Discussion

To date, a considerable amount of data has suggested that vitamin D and B9 deficiency may contribute to depressive and anxiety disorders, for which neuroticism is a strong predictor. On this basis, we expected to find negative correlations between neuroticism and PGS for these vitamins in healthy individuals from the Russian population. In general, this hypothesis was not confirmed. Instead, we found sex differences in the studied relationships. Women demonstrated a negative correlation of neuroticism with the VB9-PGS, no relationship with the VD-PGS, and a significant positive correlation with the N-PGS. Overall, these data suggest that in women, low vitamin B9 levels may contribute to a higher expression of this trait in addition to the genetic predisposition to neuroticism captured by N-PGS, whereas vitamin D levels do not significantly impact emotional instability. In men, in contrast, VB9-PGS did not affect neuroticism. VD-PGS correlated with neuroticism but in the opposite direction to the expected one: higher VD-PGS corresponded to higher neuroticism scores, and this was observed mainly in men with high extraversion. In addition, in men, unlike women, neuroticism was not significantly related to N-PGS.

The sex-related pattern of the relationship between neuroticism and genetic liability to higher vitamin D and B9 concentrations might have been expected given the well-known sex differences in neuroticism and vitamin metabolism. Women usually demonstrate higher neuroticism scores than men, with inconsistent data for the angry hostility facet (1, 55). With respect to neuroticism factors, women were found to have higher scores on the Withdrawal factor, whereas Volatility scores were higher in men than in women, at least among White participants (56). A GWAS has revealed sex differences in the genetic background of neuroticism, with enrichment of genes expressed in the pituitary gland in men (12). In addition, studies have indicated an incomplete overlap of genetic variants associated with different components of neuroticism (11, 55) and sex- and item/factor-specific genetic correlations of neuroticism with other traits (11, 12, 55). Notably, a weak negative genetic correlation between neuroticism and vitamin D has reached significance in women but not in men (12). There is also evidence of sex differences in vitamin D bioavailability and metabolism and health consequences of its deficiency, with a higher serum 25(OH)D concentration in men, which may be due to the regulation of gene expression by sex hormones, the amount and function of adipose tissue, and behavior factors (57).

In line with our data, serum folate concentrations have been shown to correlate negatively with depression in women but not in men in a large population-based sample (58). Several potential mechanisms might link folate with mood regulation. These include folate’s role in the biosynthesis of monoamine neurotransmitters via effects on S-adenosylmethionine or by enhancing synthesis of, or substituting for tetrahydrobiopterin (59). At the same time, animal models suggest involvement of the estradiol/ERβ/PI3K/AKT signaling pathway in the relationship between folate deficiency and depression-like behavior in females but not males (60).

In contrast to the sex differences, the directionality of the VD-PGS effect on neuroticism was unexpected and contradicted the hypothesis. As we found positive correlations between VD-PGS and PGS for bipolar disorder type 1 and PGS for alcohol use disorders (resembling volatility traits) but not with MDD-PGS or BD2-PGS (resembling withdrawal traits), one explanation might be horizontal pleiotropy, i.e., an independent action of the same genes/polymorphisms on vitamin D concentration and on excitability-related traits, the genetic background of which are poorly captured by the N-PGS used in the present study. Other scenarios are also possible, including one suggesting that the link between VD-PGS and excitability-related aspects of neuroticism might indeed be mediated by vitamin D status. There is some support for this hypothesis from animal and human studies. A vitamin D-enhanced diet has been shown to reduce startle response habituation in juvenile mice (61). In humans, vitamin D stimulates the production of BDNF (62), which is positively correlated with neuroticism (63). Vitamin D enhances the expression of tyrosine hydroxylase, the rate-limiting enzyme in the synthesis of catecholamines, which play an important role in sympathetic nervous system activity (64). These data are in accordance with Eysenck’s model (52), in which increased activity of the sympathetic nervous system is a physiological basis of high neuroticism. Notably, however, the relationships between serum vitamin D levels, norepinephrine, and sympathetic nervous system reactivity are quite complex and poorly studied (65).

The sex-determined heterogeneity of neuroticism at the phenotypic and genetic levels cannot fully explain the contradiction of our data with those of a previous study that revealed a negative association of VD-PGS with neuroticism (38). By statistically controlling for sex, Avinun et al. (38) reported negative correlations of VD-PGS with all aspects of neuroticism; though, unlike shyness, anxiety, hostility, and vulnerability, correlations with impulsivity and depression were not significant. One reason for the discrepancy between these and our results may be the use of different neuroticism measures (Eysenck’s model versus the Big Five model) and different GWASs for computing VD-PGS. Our analysis was based on a later and larger GWAS (34) than that (66) used by Avinun et al. (38). Furthermore, interactions of genotype with the environment, specifically the availability of ultraviolet exposure, can explain the contradictory results. Our sample living at 55° north latitude and that of Avinun et al. (38) recruited through Duke University (36° north latitude) likely differ in ultraviolet exposure.

The lack of data on other aspects of the region-specific environmental contexts is one of the limitations of our investigation and, at the same time, emphasizes the need for national studies on the link between vitamins, neuroticism, and health risks. Another limitation is the lack of information on individual lifestyle factors that may influence vitamin status and the unknown actual vitamin status of the participants due to the retrospective nature of the study. It should be noted, however, that at the time of recruitment none of the participants suffered from diseases limiting physical activity or had an overweight sufficient to diagnose obesity. Importantly, none of the participants had first-degree relatives with affective disorders. Future studies can explore the pleiotropy issue via a mediation analysis of the influence of VD-PGS on neuroticism, with the vitamin’s serum concentration as a mediating factor. Next, given that some sex differences between the effects studied did not reach significance, the issue of power should be considered. The whole sample size provided an acceptable power (75%: G*Power: hierarchical regression with 6 independent variables) to detect the effect of *R*^2^ = 0.02 previously reported for VD-PGS (38). At the same time, regarding the auxiliary analyses, the small sample size was a limitation of the study, especially when temperament subgroups were considered. Finally, the source of the significant difference in the VD-PGS between men and women in our study is not clear, as the sex-related factors influencing vitamin D bioavailability and higher serum concentrations of vitamin D in men (22, 57) could not directly explain this difference.

## 5 Conclusion

The results suggest that the effects on neuroticism of genetic propensity for suboptimal vitamin D and B9 concentrations might differ across the two sexes. The findings are consistent with the idea of the importance of vitamin B9 for emotional stability in women and indicate the involvement of genetic factors predisposing to higher vitamin D levels in excitability-related components of neuroticism in men. Thus, the findings do not provide support for the hypothesis that increasing vitamin D levels can reduce neuroticism. Furthermore, they caution that in some cases, excess vitamin D may be associated with lower emotional stability in men. Future research is needed to characterize the impact of interplay between genetic liability to lower and higher vitamin D levels and region-specific factors related to vitamin availability, such as diet and outdoor activity, on neuroticism using actual vitamin levels as mediators. At that, future studies should provide a more comprehensive assessment and separate analysis of patterns in groups of men and women.

## Data Availability

The data analyzed in this study is subject to the following licenses/restrictions: The raw data supporting the conclusions of this article will be made available by the authors upon request with the permission of the Biomaterial Collection Fund “NeuroResource”. Requests to access these datasets should be directed to Margarita Alfimova, m.alfimova@gmail.com.
